# Coordination of Respiration, Swallowing, and Chewing in Healthy Young Adults

**DOI:** 10.3389/fphys.2021.696071

**Published:** 2021-07-13

**Authors:** Naohito Hao, Anna Sasa, Sirima Kulvanich, Yuta Nakajima, Kouta Nagoya, Jin Magara, Takanori Tsujimura, Makoto Inoue

**Affiliations:** Division of Dysphagia Rehabilitation, Niigata University Graduate School of Medical and Dental Sciences, Niigata, Japan

**Keywords:** electromyography, mastication, respiration, suprahyoid muscles, swallowing

## Abstract

Examining the coordination of respiration and swallowing is important for elucidating the mechanisms underlying these functions and assessing how respiration is linked to swallowing impairment in dysphagic patients. In this study, we assessed the coordination of respiration and swallowing to clarify how voluntary swallowing is coordinated with respiration and how mastication modulates the coordination of respiration and swallowing in healthy humans. Twenty-one healthy volunteers participated in three experiments. The participants were asked to swallow 3 ml of water with or without a cue, to drink 100 ml of water using a cup without breathing between swallows, and to eat a 4-g portion of corned beef. The major coordination pattern of respiration and swallowing was expiration–swallow–expiration (EE type) while swallowing 3 ml of water either with or without a cue, swallowing 100 ml of water, and chewing. Although cueing did not affect swallowing movements, the expiratory time was lengthened with the cue. During 100-ml water swallowing, the respiratory cycle time and expiratory time immediately before swallowing were significantly shorter compared with during and after swallowing, whereas the inspiratory time did not differ throughout the recording period. During chewing, the respiratory cycle time was decreased in a time-dependent manner, probably because of metabolic demand. The coordination of the two functions is maintained not only in voluntary swallowing but also in involuntary swallowing during chewing. Understanding the mechanisms underlying respiration and swallowing is important for evaluating how coordination affects physiological swallowing in dysphagic patients.

## Introduction

Swallowing is essential not only to propel a food bolus into the stomach but also to protect the upper airway and prevent pulmonary aspiration. During swallowing, several mechanisms function simultaneously; the tongue and soft palate elevate to propel the bolus posteriorly and open the fauces, respectively, accompanied by the elevation of the hyolaryngeal complex and pharyngeal muscle contraction to transport the bolus, laryngeal closure to serve as airway protection, and finally, relaxation of the upper oesophageal sphincter to allow the bolus to enter the esophagus (Jean, 2001). Most of these structures also serve as a common pathway for other behaviors, such as mastication, respiration, and vocalization.

Breathing and swallowing are tightly coupled to perform safe and effective coordination (Martin-Harris and McFarland, 2013). Both the respiratory and swallowing neural networks, which generate and coordinate muscle activities, are located in the lower brainstem (Amri et al., 1984; Bianchi and Gestreau, 2009). Previous studies have suggested that within the swallowing neural network in the brain stem, some neurons, such as interneurons and motor neurons, may also participate in the respiratory activity. The respiratory central pattern generator (CPG) consists of the dorsal respiratory group and the ventral respiratory column in the medulla and pneumotactic centers in the pons (Bianchi and Gestreau, 2009). In contrast, the swallowing CPG is located in the medulla oblongata and includes a dorsal swallowing group (DSG) in the nucleus tractus solitarius and a ventral swallowing group (VSG) within the ventrolateral medulla above the nucleus ambiguous (Jean, 2001). Both CPGs receive afferent sensory inputs from the pharynx and larynx *via* the vagal nerves (Bianchi and Gestreau, 2009). Furthermore, ambiguous, trigeminal, and hypoglossal motoneurons are activated in both situations (Sumi, 1963; Larson et al., 1994; Shiba et al., 1999), which indicates that these motoneurons may receive inputs from these CPGs. Thus, it can be assumed that there is a substantial amount of cross talk between them.

Regarding coordination of respiration and swallowing in animals, swallowing reflexes are reported to operate during the different phases of respiration. Doty and Bosma (1956) first demonstrated that most swallowing evoked by the superior laryngeal nerve stimulation occurred during the inspiratory phase in anesthetized dogs and monkeys. In addition, Kawasaki et al. (1964) reported that spontaneous swallowing occurred during the inspiratory phase in anesthetized dogs. In contrast, in awake animals, Feroah et al. (2002) reported that around half of spontaneous swallows occurred during the inspiratory phase (45%) followed by the expiratory phase (28%) or the transition from expiration to inspiration (26%) in awake goats. McFarland et al. (McFarland and Lund, 1993) reported that more than half of swallowing reflexes were recorded during the first half of the inspiratory phase in awake rabbits. Several studies reported that the occurrence of the swallowing reflex coincided with the expiratory phase in anesthetized cats (Doty and Bosma, 1956; Dick et al., 1993).

In humans, most swallows occur during the expiratory phase (Nishino et al., 1985; Smith et al., 1989; McFarland et al., 1994). While most swallows are readily evoked by pharyngeal or laryngeal stimulation, humans are thought to be the only species that can voluntarily initiate swallowing. Thus, it is impossible to evaluate how the coordination of respiration and swallowing is achieved during voluntary swallowing in animal studies. Previous studies have investigated the coordination of respiration and swallowing during voluntary swallowing (Shaker et al., 1992; Martin-Harris et al., 2003; Ouahchi et al., 2019). Shaker et al. (1992) investigated the coordination of respiration and voluntary swallowing in healthy young and older adult volunteers. In both groups, the pre- and post-respiratory phase was most commonly found to be the expiratory phase, which was more frequent during 5-ml water swallowing compared with during dry swallowing. The authors also demonstrated that tachypnoea resulted in an increase in the occurrence rate of EE-type swallowing. Shaker et al. (1992); Martin-Harris et al. (2003), Ouahchi et al. (2019) reported that the most frequent pattern during 5-ml water swallowing was the EE type, although the temporal pattern of events, such as bolus transport, hyoid excursion, laryngeal closure, or opening of the upper oesophageal sphincter varied among subjects. The swallowing task in these studies was the command swallow task, in which the subject was instructed to swallow when the examiner presented a cue (Nagy et al., 2013; Kurosu et al., 2020). Nagy et al. (2013) conducted a videofluoroscopy (VF) study in healthy adults to compare swallowing behavior between cued and non-cued 10-ml water swallowing. They found that the pharyngeal transit time was significantly longer and that the bolus was more distally advanced in the pharynx at swallowing initiation with non-cued swallowing compared with cued swallowing. These results suggest that command swallowing may modulate swallowing physiology.

Coordination of respiration and swallowing during mastication should also be considered, because the food bolus must be transported into the esophagus safely during swallowing to protect the upper airway. Previous studies have reported the modulation of respiratory rhythm as well as coordination of respiration and swallowing during chewing in healthy humans (Smith et al., 1989; McFarland and Lund, 1995; Palmer and Hiiemae, 2003; Matsuo et al., 2008). Although these previous studies have demonstrated that most swallowing was evoked during the expiratory phase, some studies have produced conflicting results. Palmer and Hiiemae (2003) recorded electromyography (EMG) activity and VF images during solid food chewing, reporting that the respiratory cycle time changed between individuals and was significantly longer during banana chewing than cookie chewing. The authors suggested that bolus aggregation and hypopharyngeal transport occurred during the expiratory plateau phase, which seems to be one of the reasons for the prolonged respiratory cycle time. These results were, however, inconsistent with the findings of several other studies. Smith et al. (1989) reported no difference in respiratory cycle time, such as expiratory and inspiratory cycle time, among resting, solid food swallowing, and water drinking. Matsuo et al. (2008) reported a decrease in respiratory cycle time during chewing. Thus, the way in which the respiratory cycle is affected during chewing and its underlying mechanisms remains unclear.

In this study, we assessed the coordination of respiration and swallowing in healthy humans to clarify how cueing, drinking, and mastication modulate the coordination of respiratory and swallowing in healthy humans. For this purpose, we recorded EMG activity in the masseter (Mas, jaw closer), suprahyoid (Supra, hyoid elevator), and infrahyoid muscles (Infra, thyroid elevator) to evaluate chewing and swallowing movements and nasal temperature to monitor breathing as well as videoendoscopic (VE) images. We hypothesized that there would be a difference in swallowing physiology between cued and non-cued voluntary swallowing, and that chewing or drinking would reduce the respiratory cycle time during chewing to compensate for the narrowing of the pharyngeal cavity.

## Materials and Methods

### Participants

In this study, 21 healthy volunteers (10 men, 11 women) whose age ranged from 22 to 35 years [average age ± standard deviation (SD), 28.4 ± 3.7 years] participated. Before data collection, a dentist (NH) confirmed that all the participants had no abnormalities or temporomandibular disorders, occlusal abnormalities, masticatory problems, or swallowing problems. No participant had a history of alimentary, pulmonary, or neurological disease, structural or speech disorders, or voice problems. Informed consent was obtained from all the participants after receiving information about the experimental procedure. This study was approved by the Ethics Committee of the Niigata University Graduate School of Medical and Dental Sciences (2020-0131). The experiments were performed in accordance with the Declaration of Helsinki (2008) for humans.

### Recordings

Surface EMGs were recorded from both the right and left Mas muscle, right Supra muscles, and left Infra muscles. Electrodes (ZB-150H; Nihon Kohden, Tokyo, Japan) were attached to the skin over the center of the Mas, the anterior belly of the right digastric muscle for Supra, and the center of the left thyrohyoid muscle for Infra with an inter-electrode distance of 2 cm. EMG signals were filtered and amplified (high pass, 60 Hz) (WEB-1000; Nihon Kohden, Tokyo, Japan). A thermo-sensor system (MLT415 and ML309; Nihon Kohden, Tokyo, Japan) was used to record nasal temperature during respiration. A nasal probe was attached below the external nares to measure the temperature. Increases and decreases of temperature indicated expiration and inspiration, respectively. Since there was a difference between room temperature (around 20°C) and the nasal cavity temperature, the nasal temperature gradually increased, even during breath-holding. We calculated the rate of temperature increase in one participant. At several randomly determined temperatures, the participant was asked to hold her breath for 10 s, and changes in temperature were measured (Supplementary Figure 1). Although the rate of temperature increase was negatively correlated with the initial temperature, it was relatively small compared with that during respiration.

VE images were recorded in 11 participants (six men, five women; age range from 24 to 32 years, average age ± SD, 29 ± 2.7 years) to measure the distance between the posterior wall of the pharynx and epiglottis and between the lateral walls of the pharynx in Protocol 3 (see below). A fiber-optic endoscope (FNL-10RP3; Pentax, Tokyo, Japan) was inserted through the nasal passage and into the mid pharynx.

All signals were collected *via* an interface board (PowerLab; ADInstruments, Colorado Springs, CO, United States) and stored on a personal computer. The sampling rate was 10 kHz for EMGs, 100 Hz for nasal temperature, and 33 Hz for VE images. Data synchronization and analysis were performed using the PowerLab software package (Video Module and LabChart 8; ADInstruments, Colorado Springs, CO, United States), which automatically aligned the data at different sampling rates.

### Data Recordings

The participants were instructed not to eat or drink for at least 1 h before the experiment. They were asked to sit in a chair with their head vertical to the Frankfort plane. In this study, we carried out three experiments on the same day for each individual. In the first experiment (Protocol 1), 3 ml of water was inserted into the mouth *via* a syringe; the participants were asked to keep the water on the floor of the mouth, then swallow it when prompted by a cue as quickly as possible (with cue) or whenever they wanted to (without cue) in a single swallow [dipper-type swallow (Cook et al., 1989)]. In the second experiment (Protocol 2), the participants were asked to drink 100 ml of water using a cup without breathing between swallows. In the third experiment (Protocol 3), the participants were asked to eat a 4-g portion of corned beef (Echigo Uonuma low fat corned beef, Forica Foods, Horinouchi, Japan) using a spoon in their usual manner. The time interval between trials was at least 2 min, and the participants were able to rinse their mouths with distilled water whenever they wished between the trials.

### Data Analysis

The onset and offset of EMG activity, following smoothing of the rectified EMGs (time constant 20 ms), were determined. Mean value ± SD of EMGs at rest for 5 s was obtained as a control for each participant. When the values exceeded the control + 2 SDs during the trials, the EMG burst was considered active.

Swallowing activity was evaluated by Supra and Infra EMG burst duration (time) and area. Time and area of these muscles were defined as the time interval between onset and offset, and area under the curve (area) of rectified and smoothed EMG burst, respectively. The amplitude of EMGs was normalized to the mean amplitude of rectified and smoothed EMG burst during 3-s maximum jaw closing for Mas and during 3-s maximum jaw opening for Supra and Infra.

First, we recorded the five respiratory cycles at rest for all the participants to obtain the mean respiratory cycle time, such as the expiratory and inspiratory time. Respiratory cycle time (respiratory time) was defined as the time from the start of one inspiration to the start of the next inspiration. Respiratory time was then divided into inspiratory and expiratory times. The duration of the inspiratory phase (inspiratory time) and expiratory phase (expiratory time) were defined as the time from the onset of inspiration to the end of inspiration and the time from the end of inspiration to the onset of the next inspiration, respectively. Each respiratory cycle had inspiratory and expiratory phases, sometimes with a pause after expiration. To identify each time duration, we included these short plateau phases in the expiratory phase. Furthermore, swallowing apnoea was commonly observed during swallowing and was measured in accordance with previous studies (Martin-Harris et al., 2003; Butler et al., 2004; Matsuo et al., 2008).

The swallowing pattern was determined based on the respiratory phase immediately before and after swallowing. When the onset of pharyngeal swallowing, defined as the onset of Supra EMG burst, was preceded and followed by the expiratory phase, we designated the swallow as expiration–swallow–expiration type (EE type). When the onset of swallowing was preceded by the inspiratory phase and followed by the expiratory phase, we designated the swallow as inspiration–swallow–expiration type (IE type). When the onset of swallowing was preceded by the expiratory phase and followed by the inspiratory phase, we designated the swallow as expiration–swallow–inspiration type (EI type). When the onset of swallowing was preceded by and followed by the inspiratory phase, we designated the swallow as inspiration–swallow–inspiration type (II type). Previous studies have defined other criteria, such as swallows occurring at the transition between inspiration and expiration, which were designated inspiratory–expiratory (I–E) transition swallows, and swallows occurring at the transition between expiration and the inspiratory phase of the next breath, which were designated expiratory–inspiratory (E–I) transition swallows (Kijima et al., 1999). In this study, we did not use these terms, because we intended to simplify the pattern of coordination between respiration and swallowing.

In Protocol 1, the mean values of Supra and Infra time and area were compared between with and without cue conditions. Next, the occurrence frequency of coordination between respiration and swallowing (EE, IE, EI, or II type) was compared between with and without cue conditions. For the major pattern of coordination, changes in respiratory time, such as inspiratory and expiratory time, were compared among before, during, and after swallowing. It can be assumed that the coordination pattern was dependent on the timing of cueing. Therefore, the onset time of the expiratory phase was defined as the reference time “0,” and the time of cueing, onset, and offset of swallowing determined by the Supra EMG burst was calculated in the “with cue” session.

In Protocol 2, the coordination pattern was determined by the same procedure in Protocol 1. During 100-ml water swallowing, breath-holding must be performed until drinking is finished. We determined whether the swallowing apnoea time affected the coordination pattern in this protocol. Next, changes in respiratory time, such as inspiratory and expiratory time, were compared among before, during, and after swallowing.

In Protocol 3, we selected the data of masticatory sequence until the first swallow for analysis. The number of respiratory cycles and respiratory time, such as inspiratory and expiratory time of each respiratory cycle, were measured during mastication, and the coordination pattern of respiration and swallowing was determined. Respiratory, inspiratory, and expiratory times were compared between the conditions; at rest vs. during chewing. These times were also compared between the first and last respiratory cycles. In this session, changes in airway size at the pharynx during chewing were evaluated using VE images (Supplementary Figure 2). Since we did not measure the real size on the endoscopic view, the width of the epiglottis was determined as a reference. The normalized distance between the right and left walls of the oropharynx (width) and between the posterior wall of the oropharynx and posterior edge of the epiglottis (A–P distance) was measured three times at the inspiratory–expiratory and expiratory–inspiratory transition at rest, and mean values were obtained for each participant. Width and A–P distance were also measured at the third peak of Mas EMG burst and Supra EMG burst, and at the third last peak of Mas EMG burst and Supra EMG burst during chewing.

All the data were analyzed by two experts (NH and AS). To estimate the inter-rater reliability, the intraclass correlation coefficient (ICC) was obtained, and the average was used for the subsequent analysis. Statistical analyses were performed using the SigmaPlot software (SigmaPlot 14.0, Systat Software Inc., San Jose, CA, United States) and Bell Curve for Excel (Social Survey Research Information Co., Ltd., Tokyo, Japan). Tests for normality and equality of variances were initially performed, followed by a paired *t*-test or Wilcoxon signed rank test to analyze differences between two paired groups, or by a *t*-test or Wilcoxon’s rank-sum test to analyze differences between two groups. Further, for multiple comparisons, we performed a one-way repeated measures analysis of variance (ANOVA) or one-way repeated measures ANOVA on ranks according to the results of test for normality. This was followed by Tukey’s test. The relationship between the two data sets was evaluated using Spearman’s rank order correlation. *P*-values < 0.05 were considered significant.

## Results

### Reliability of the Data

The ICCs for parameters were calculated (Table 1) and were significant, although the ICCs of EMG data were relatively small. This was expected, because the threshold differed between the raters. Of the participants in the third experiment (Protocol 3), the VE data for the width of the pharynx were excluded in two participants because the whole view of the pharynx was not observed.

**TABLE 1:** Intraclass correlation coefficients.

		***F* value**	**ICC**	***P* value**
EMG	Supra time	3.6083	0.7229	*P* < 0.001
	Infra time	3.6259	0.7242	*P* < 0.001
Respiration	Insp time	21.2420	0.9529	*P* < 0.001
	Exp time	12.3628	0.9191	*P* < 0.001

*EMG, electromyography; Exp time, expiratory time; ICC, intraclass correlation coefficient; Infra time, infrahyoid electromyographic burst duration; Insp time, inspiratory time; Supra time, suprahyoid electromyographic burst duration.*

### Protocol 1: 3-ml Water Swallowing

**FIGURE 1:**
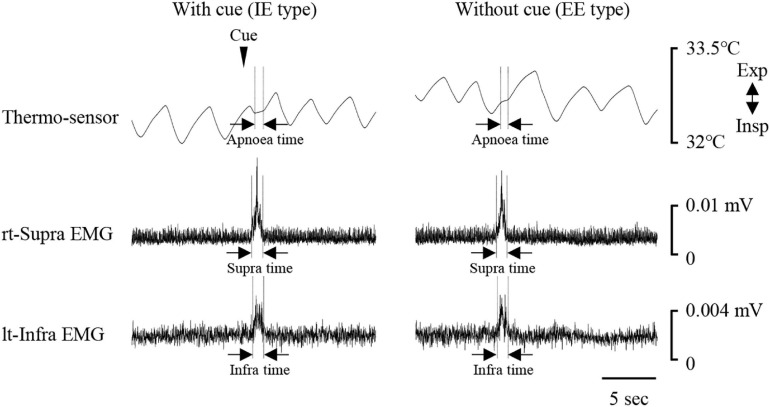
Examples of respiratory and swallowing recordings during 3-ml water swallowing with (left) and without cue (right). Electromyography (EMG) waveforms were rectified and smoothed. Periods of swallowing apnoea and EMG burst are shown by dotted lines. The coordination pattern was inspiration–swallow–expiration (IE) with cue (left), and expiration–swallow–expiration (EE) without cue. The timing of cueing is indicated by an arrowhead. lt-Infra, left infrahyoid, rt-Supra, right suprahyoid.

**FIGURE 2:**
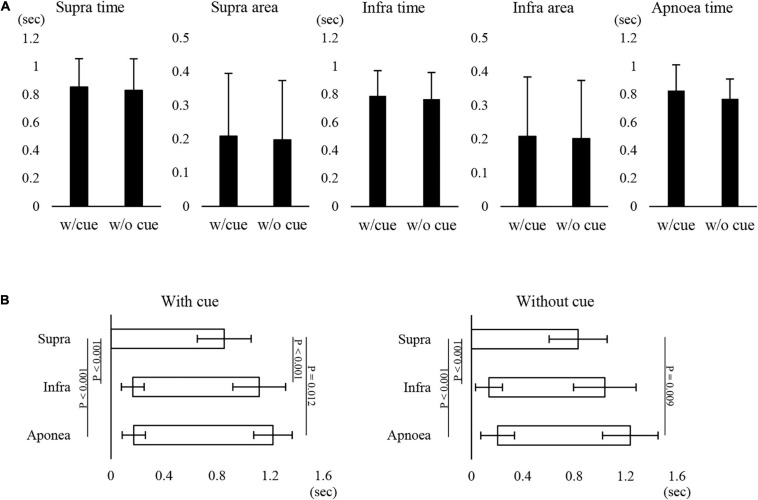
Effect of cueing on 3-ml water swallowing behaviors in all cases. **(A)** There were no differences in the suprahyoid (Supra) electromyography (EMG) burst duration (Supra time) or area (Supra area), infrahyoid (Infra) EMG burst duration (Infra time) or area (Infra area), or swallowing apnoea time between with (w/cue) and without cue (w/o cue) (paired *t*-test). **(B)** In with (left) and without cue (right) conditions, the onset and offset of Supra EMG burst were followed by those of Infra EMG burst and swallowing apnoea (Apnoea) (one-way repeated measures ANOVA followed by Tukey test for onset with cue, *P* < 0.001, df = 2, *F* = 51.866; one-way repeated measures ANOVA on ranks followed by Tukey test for offset with cue, *P* < 0.001; one-way repeated measures ANOVA on ranks followed by Tukey test for onset without cue, *P* < 0.001; one-way repeated measures ANOVA on ranks followed by Tukey test, *P* < 0.001). The results of *post hoc* test are shown in the figure.

**FIGURE 3:**
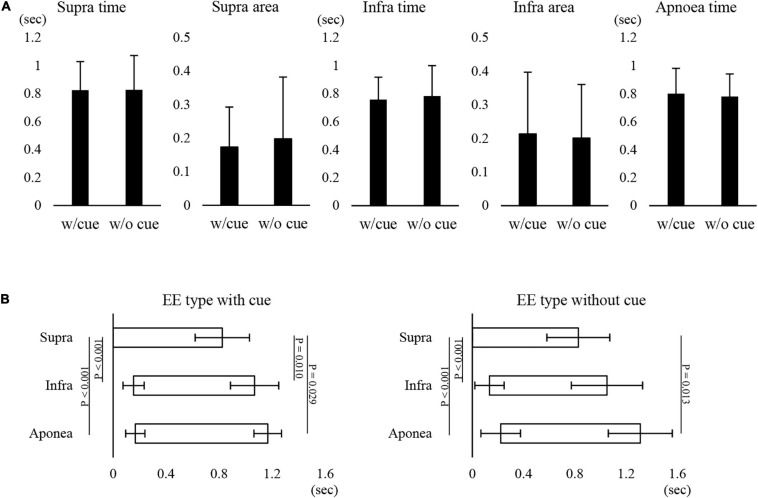
Effect of cueing on 3-ml water swallowing behaviors in EE type. **(A)** There were no differences in the suprahyoid (Supra) electromyography (EMG) burst duration (Supra time) or area (Supra area), infrahyoid (Infra)EMG burst duration (Infra time) or area (Infra area), or swallowing apnoea time between with (w/cue) and without cue (w/o cue) (Student’s *t*-test). **(B)** In the with (left) and without cue (right) conditions, onset and offset of Supra EMG burst were followed by those of Infra EMG burst and swallowing apnoea (Apnoea) (one-way repeated measures ANOVA followed by Tukey test for onset with cue, *P* < 0.001, df = 2, *F* = 34.876; one-way repeated measures ANOVA on ranks followed by Tukey test for offset with cue, *P* = 0.004; one-way repeated measures ANOVA followed by Tukey test for onset without cue, *P* < 0.001, df = 2, *F* = 14.624; one-way repeated measures ANOVA on ranks followed by Tukey test, *P* = 0.009).

Figure 1 shows examples of raw data of recordings. In both the “with cue” and “without cue” conditions, the most frequent coordination pattern was EE type (Table 2). We evaluated how quickly the participants responded to cueing to initiate swallowing. There was no difference in the time from cueing to onset of Supra EMG burst between the coordination patterns (*t*-test); 0.83 ± 0.37 s (*n* = 15) for EE type vs. 0.7 ± 0.2 s (*n* = 6) for IE type. EMG duration time and area, as well as swallowing apnoea time, were no different between the “with cue” and “without cue” conditions (Figure 2A). This was also the case in the temporal pattern of muscle activity, in that the Supra EMG bursts always preceded the Infra EMG burst and swallowing apnoea in both cases (Figure 2B). Since the major pattern of coordination was EE type, we also compared those parameters for EE type between the “with cue” and “without cue” conditions. These findings were consistent with the overall results (Figure 3). These results indicated that command swallowing did not change the swallowing pattern, at least when swallowing 3 ml of water.

**TABLE 2:** Coordination pattern of respiration and swallowing.

	**EE**	**IE**	**EI**	**NA**
3-ml water with cue	15	6	0	0
3-ml water without cue	15	4	2	0
100-ml water	11	3	2	5
Chew and swallow	14	1	1	5

*EE, expiration–swallow–expiration type; EI, expiration–swallow–inspiration type; IE, inspiration–swallow–expiration type. NA indicates cases of subjects who failed to swallow 100 ml with breath holding for the 100-ml water swallowing task and the case of subjects who held their breath while chewing in the chew and swallow task.*

**FIGURE 4:**
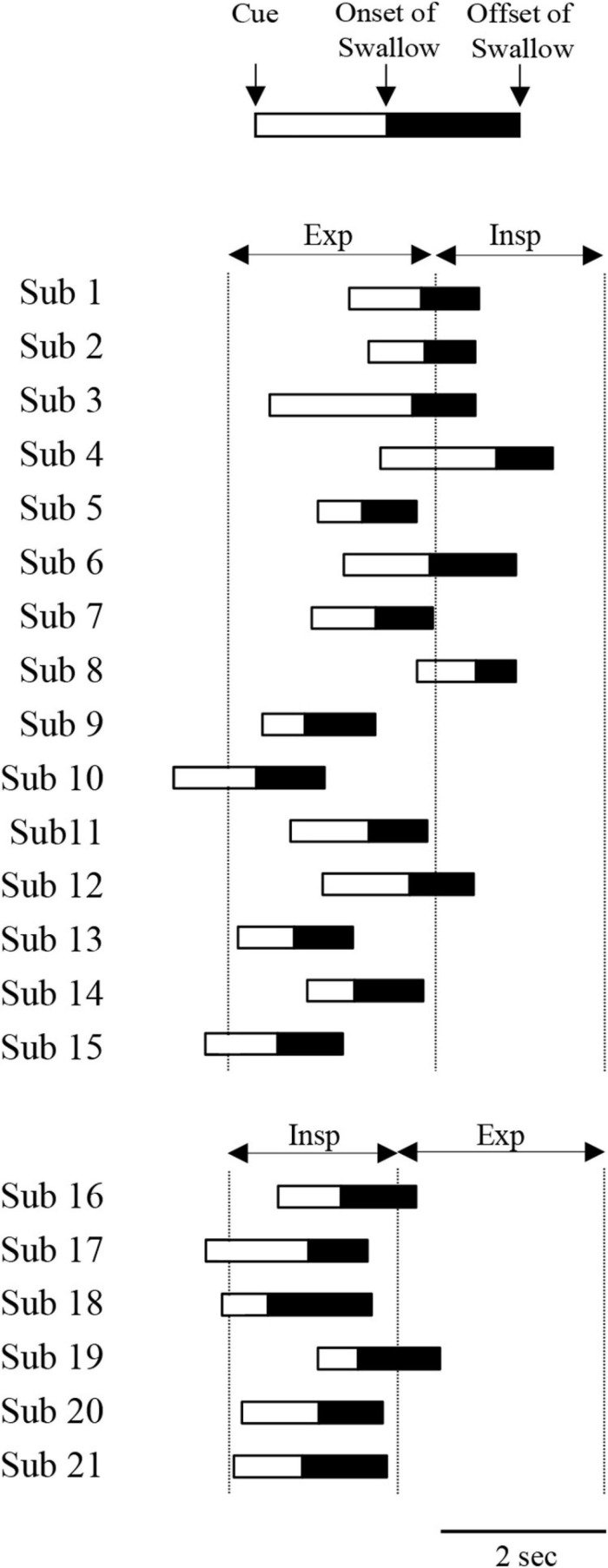
Time of cuing, onset and offset of suprahyoid electromyography (EMG) burst during 3-ml water swallowing with cue. Participants 1–15 and 16–21 exhibited expiration–swallow–expiration (EE) type and inspiration–swallow–expiration (IE) type, respectively. The time was aligned with the onset of the expiratory phase for EE type and the onset of the inspiratory phase for IE type. Exp and Insp indicate the mean duration of expiratory and inspiratory phases at rest, respectively.

Next, we investigated why the most frequent pattern of coordination was EE type in both the “with cue” and “without cue” conditions. In this study, the mean respiratory cycle time was 4.7 ± 1.4 s (*n* = 21), and the inspiration–expiration ratio (IE ratio) was 1:1.63 (*n* = 21). In EE type with cue, the cue was provided from the last stage of inspiratory phase to the expiratory phase (Figure 4). In contrast, the cue was given from the last stage of expiratory phase to the inspiratory phase in IE type (Figure 4). It was likely that the coordination pattern was dependent on the timing of the cue. EE type was the most frequent without cue as well as with cue. However, the subset of participants who showed EE type with cue was not identical to the subset of those who showed EE type without cue. Comparing the ratio of expiratory time to the respiratory time between the participant group in EE and IE types without cue revealed no difference; 0.62 ± 0.06 in EE type (*n* = 15) vs. 0.58 ± 0.04 in IE type (*n* = 4).

**FIGURE 5:**
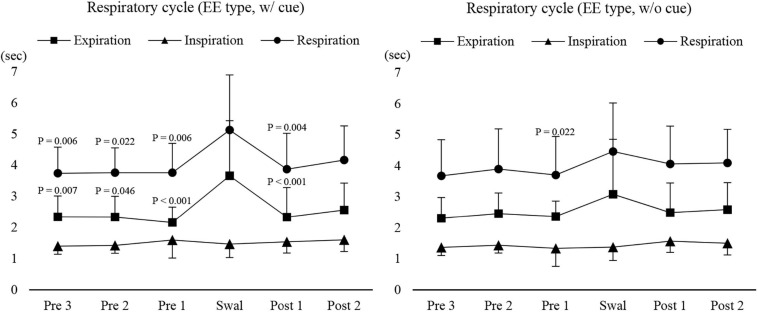
Changes in respiratory, inspiratory, and expiratory time before, during, and after 3-ml water swallowing (expiration–swallow–expiration type, EE type) with (w/cue) and without cue (w/o cue). Swallowing apnoea time was excluded. With cue, the expiratory time was significantly longer during swallowing (Swal) compared with before (Pre) and after (Post) swallowing (one-way repeated measures ANOVA on ranks followed by Tukey test, *P* < 0.001). This was also the case for the respiratory time, which was significantly longer during swallowing compared with before (Pre 1–3) and after (Post 1) swallowing (one-way repeated measures ANOVA on ranks followed by Tukey test, *P* < 0.001). The results of *post hoc* test are shown in the figure. Without cue, there was a significant difference only between before (Pre 1) and during swallowing (Swal) for the respiratory time (one-way repeated measures ANOVA on ranks followed by Tukey test, *P* = 0.025).

Next, we compared the coordination between respiration and swallowing in the group with EE type between with and without cue. While the inspiratory time was not affected by cueing, the respiratory cycle time and expiratory time were significantly longer during swallowing compared with those before and after swallowing only during 3-ml swallowing with cue, while there was no difference in any of the times without cue (Figure 5). These results suggest that the modulation of respiratory cycle time was affected by command.

Based on these results, we speculate that not only the difference in the time duration between the inspiratory and expiratory phases but also other factors may be responsible for determining the coordination pattern of respiration and swallowing.

### Protocol 2: 100-ml Water Swallowing

**FIGURE 6:**
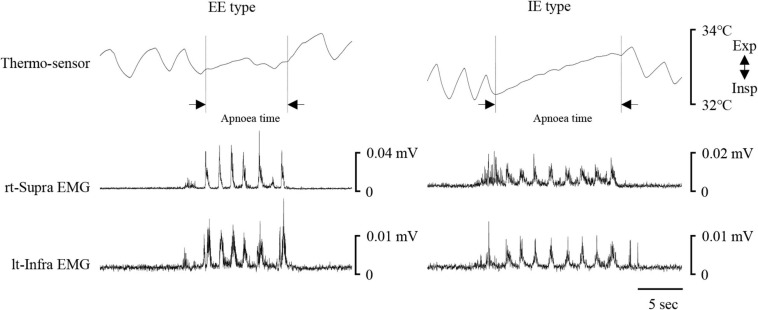
Examples of respiratory and swallowing recordings during 100-ml water swallowing. Electromyography (EMG) waveforms were rectified and smoothed. Left and right cases show expiration–swallow–expiration (EE) type and inspiration–swallow–expiration (IE) type, respectively. Although the participants held their breath during swallowing, nasal temperature gradually increased, possibly because of difference between nasal temperature and air temperature of the experimental room. The period of swallowing apnoea (Apnoea time) is shown by dotted lines. lt-Infra, left infrahyoid, rt-Supra, right suprahyoid.

Figure 6 shows examples of raw data of recordings. Of all the participants, five could not complete the 100-ml swallowing with breath-holding task, so their data were excluded from analysis. As in the 3-ml water swallowing task, the most common coordination pattern was EE type (Table 2). The total number of swallows was 8.8 ± 2.5 swallows, and the duration of swallowing apnoea was 10.7 ± 3.2 s (*n* = 16). The results indicated that it was unlikely that swallowing apnoea time was associated with the coordination type (10.9 ± 3.4 s for EE type, *n* = 10; 9 ± 1.3 s for IE type, *n* = 3; 11.5 ± 4 s for EI type, *n* = 3). The mean Supra and Infra areas per swallow were not significantly different from those during 3-ml water swallowing; 0.2 ± 0.13/swallow (*n* = 16) during 100-ml swallowing vs. 0.21 ± 0.19/swallow during 3-ml swallowing with cue (*n* = 21) for Supra area, and 0.21 ± 0.18/swallow (*n* = 16) during 100 ml swallowing vs. 0.3 ± 0.21/swallow during 3-ml swallowing with cue (*n* = 21) for Infra area, respectively (Mann–Whitney Rank Sum Test).

**FIGURE 7:**
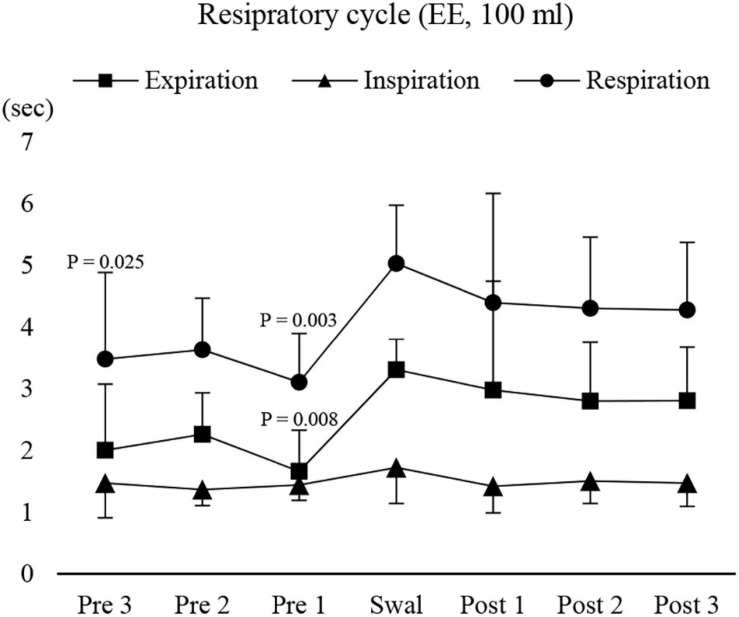
Changes in respiratory, inspiratory, and expiratory time before, during, and after 100-ml water swallowing (expiration–swallow–expiration type, EE type). Swallowing apnoea time was excluded. Expiratory time immediately before (Pre 1) swallowing was significantly shorter than that during swallowing (Swal) (one-way repeated measures ANOVA followed by Tukey test, *P* = 0.005, df = 5, *F* = 3.887). This was also the case for the respiratory time, which was significantly shorter before (Pre 1 and 3) swallowing compared with that during swallowing (one-way repeated measures ANOVA followed by Tukey test, *P* = 0.005, df = 5, *F* = 4.39). The results of the *post hoc* test are shown in the figure. Post, after swallowing.

We then investigated the modulation of respiratory movements. The respiratory cycle time and expiratory time immediately before swallowing were significantly shorter than those during swallowing (Figure 7). Inspiratory time was no different throughout the recording period. These results suggest that modulation of respiratory cycle time occurred before swallowing by shortening the expiratory time.

### Protocol 3: Chewing and Swallowing

**FIGURE 8:**
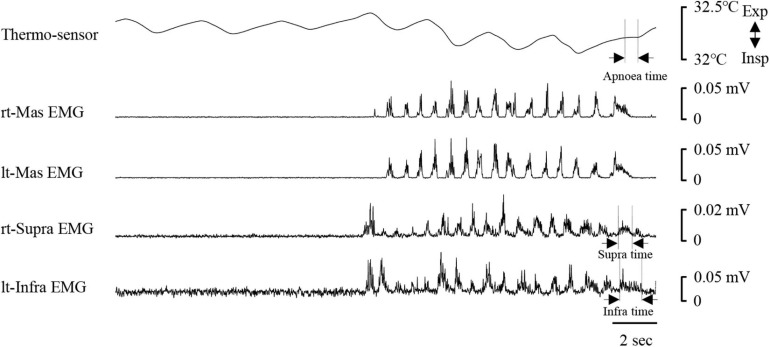
Example of respiratory and electromyography (EMG) recordings during chewing. EMG waveforms were rectified and smoothed. Note that the respiratory cycle time decreased once mastication started. The coordination pattern of respiratory and swallowing was expiration–swallow–expiration type. Periods of swallowing apnoea (Apnoea time) and swallow-related EMG burst (suprahyoid EMG time duration, Supra time; infrahyoid EMG time duration, Infra time) are shown by dotted lines. rt-Mas, right masseter; lt-Mas, left masseter; rt-Supra, right suprahyoid; lt-Infra, left infrahyoid.

**FIGURE 9:**
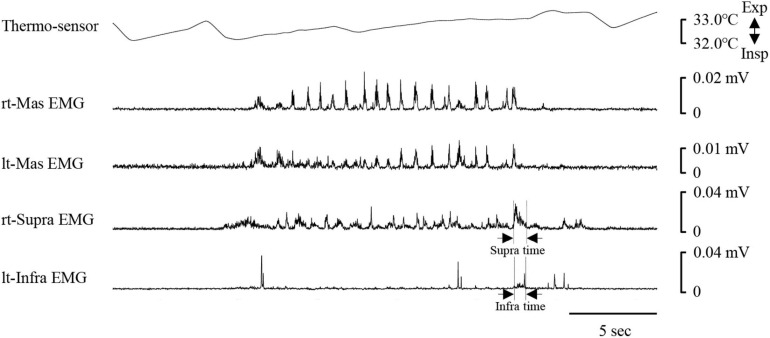
Example of respiratory and electromyography (EMG) recordings in one participant who inhibited her breath during chewing. In this case, no clear period of swallowing apnoea could be identified. Infra time, infrahyoid EMG burst duration; rt-Mas, right masseter; lt-Mas, left masseter; rt-Supra, right suprahyoid, lt-Infra, left infrahyoid; Supra time, suprahyoid EMG burst duration.

**FIGURE 10:**
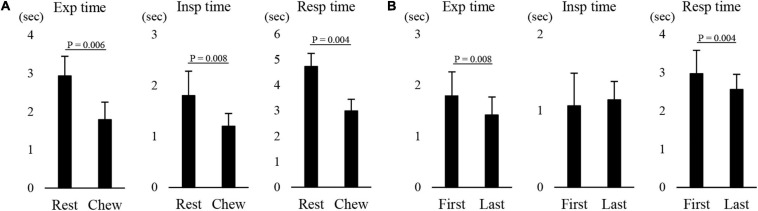
Effect of chewing on respiratory movements. **(A)** There were significant differences in the expiratory (Exp time), inspiratory (Insp time), and respiratory time (Resp time) between at rest (Rest) and during chewing (Chew) (paired *t*-test). **(B)** There was a significant difference in the Exp time and Resp time between the first and last respiratory cycles (paired *t*-test).

Figure 8 shows an example of raw data of recordings. The mean number of chewing cycles, chewing duration, and chewing cycle time until the first swallow were 17.1 ± 7.9 cycles, 11.9 ± 5.8 s, and 0.7 ± 0.11 s/cycle, respectively (*n* = 21). The number of chewing cycles and chewing duration ranged widely among the participants; the number of chewing cycles ranged from 6 to 29 cycles, and the duration ranged from 4.6 to 30 s. Of the all participants, five participants held or inhibited their breathing during chewing (Figure 9). In this group, the number of chewing cycles was 10.8 ± 4.1 cycles (*n* = 5), and the chewing duration was 8.3 ± 3.3 s (*n* = 5), which tended to be smaller compared with the whole group. We excluded those participants from the subsequent analysis. In the remaining 16 participants, the mean respiratory cycle time, expiratory time, and inspiratory time were significantly smaller during chewing compared with at rest (Figure 10A). Further, comparing them between the first and last respiratory cycles, the respiratory cycle time and expiratory time were significantly smaller at the former than the latter (Figure 10B). There was no relationship between respiratory cycle time at rest and chewing rate (cc, −0.188, *P* = 0.476), between respiratory cycle time at rest and during chewing (cc, −0.206, *P* = 0.364), and between chewing rate and respiratory cycle time during chewing (cc, −0.122, *P* = 0.640).

Since the size of the pharynx changes because of the dynamic movements of posterior tongue during chewing, we compared the width and A–P distance of the pharynx between before and during chewing. There was no difference between the conditions (Supplementary Figure 3). These results suggest that respiratory cycle time gradually decreased during chewing, which was unlikely to have been caused by changes in the size of the pharynx.

## Discussion

### Effect of Cueing on Swallowing

We intended to clarify how single swallow was affected by cueing. Therefore, we first determined the bolus volume in the preliminary experiment. We used 3-ml water in the experiment, because this amount of water was found to be suitable for all the participants to keep the bolus in the mouth. As a result, all the participants completed a 3-ml water swallowing task. As previously reported, most coordination patterns were EE type in single voluntary swallows in both the with and without cue conditions (Shaker et al., 1992; Martin-Harris et al., 2003; Ouahchi et al., 2019). In the with cue condition, although the participants were asked to swallow as quickly as possible after the cue, they may have determined the timing of swallowing in accord with the respiratory phase (i.e., the expiratory phase). In this respect, the latency was 0.83 ± 0.37 s for EE type and 0.7 s for IE type. Michou et al. (2012) also measured the fast-swallowing reaction time when participants were asked to perform swallowing of 5-ml boluses of liquids delivered into the mouth as quickly as possible while the latency between the delivery time of the electrical cue to the onset of the pharyngeal swallow was measured. The mean time is reported to be 0.886 s, which is consistent with the results of this study. We suggest that the participants in this study completed 3-ml swallowing with the cue regardless of the respiratory phase.

In this study, the mean respiratory cycle time was 4.7 ± 1.4 s, and the IE ratio was 1:1.6, which was within the normal range (Boyle, 2001). The occurrence ratio of EE type was much higher (EE type, 15 vs. IE type, 6) compared with the IE ratio (inspiration 1.6 vs. expiration 1). We found that in the “with cue” condition, most EE type swallows were initiated when cues were given from the very late inspiratory phase to the expiratory phase, while most IE type swallows were initiated when cues were given from the very late expiratory phase to the inspiratory phase. We speculated that the timing of cueing affected the coordination pattern of respiration and swallowing. However, this raises the question of why the frequency of occurrence of EE type swallows was dominant in the without cue condition. There are substantial differences in the timing of swallowing initiation across species and experimental conditions. In most mammals, such as dogs, monkeys, and rabbits, swallowing reflexes are typically evoked during the inspiratory phase (Doty and Bosma, 1956; Kawasaki et al., 1964; McFarland and Lund, 1993; Feroah et al., 2002). In contrast, as shown in this study, most swallows are evoked during the expiratory phase either involuntarily or voluntarily in humans, as in anesthetized cats (Doty and Bosma, 1956; Nishino et al., 1985; Smith et al., 1989; Dick et al., 1993; McFarland et al., 1994). One potential cause of the difference in swallowing between humans and other species is the anatomical characteristics of the location of the hyolaryngeal complex. In most non-human mammals, the hyolaryngeal complex is located in a relatively high position (Laitman and Reidenberg, 1993). Furthermore, the height of the mid-pharynx is very small, and the tongue lies within the oral cavity and never forms the posterior part of the pharynx. This unique anatomical configuration enables the epiglottis to pass up the soft palate, allowing the larynx to open directly into the nasopharynx. Thus, the food way from the oral cavity to the esophagus and the airway from the nasal cavity to the trachea are separated, which means that these animals do not need strict coordination of respiration and swallowing movements to prevent pulmonary aspiration. When a single swallow occurs during the inspiratory phase, inspiration must be interrupted by swallowing apnoea, which is followed by a shortened duration of the expiratory phase. In contrast, if a swallow is evoked during the expiratory phase, the duration of entire expiratory phase including pre and post swallowing is lengthened. In either case, the tidal volume of the post-swallow period is increased, although the latter may be more effective than the former (Nishino et al., 1985). Possible advantages of swallowing initiation during the inspiratory phase include the facilitation of bolus transport by inhalation, and minor effects of respiration after swallowing in this situation, because the shortened duration of inspiration is less effective for activating stretch receptors in the trachea or lung (Davis et al., 1956; Bartlett et al., 1976; Bradley et al., 1987). Humans, however, need to protect their airway because of the lower position of the hyolaryngeal complex, as described above. The action of the diaphragm, which is a major inspiratory muscle, and the Infra muscles, which are all inspiratory muscles, appear to be counteracted by contraction of the Supra muscles during swallowing, at least in humans (McFarland et al., 1994).

The results revealed no significant difference in swallowing movements in terms of Supra and Infra EMG time and area, as well as swallowing apnoea, which represents pharyngeal swallowing between the with and without cue conditions. The temporal features of swallowing events were also similar to each other. These results suggest that voluntary swallowing of a small amount of bolus does not affect swallowing motor actions, regardless of swallowing command and type of coordination of respiration and swallowing. Several previous studies have compared swallowing behaviors between cued and non-cued swallows (Daniels et al., 2007; Nagy et al., 2013). Nagy et al. (2013) compared 10-ml liquid swallows between cue and non-cue conditions. When bolus transport was measured using 10 ml of ultrathin barium by VF, the results revealed that during cued swallowing, bolus advancement to the pyriform sinuses prior to swallow initiation was seen significantly less frequently, and pharyngeal transit time and response time were both significantly longer than those during non-cued swallowing (Nagy et al., 2013). Considering the difference in the effect on swallowing movements, we evaluated only Supra and Infra EMG activities. Although Supra EMG activity during swallowing represents pharyngeal swallowing, with a previous study having reported a significant linear relationship between the passage of the bolus tail at the upper oesophageal sphincter and offset of the Supra EMG burst (Tsukada et al., 2009), we only recorded muscle function and not bolus passage. In addition, the bolus volume was different among previous studies. A small amount of water (3 ml) might cause a failure to modulate swallowing physiology. Furthermore, Nagy et al. (2013) did not measure the response latency to swallowing initiation. If the participant had responded to cueing more quickly than the participants in this study, bolus transport would be expected to be affected.

We did not expect to find a difference in respiratory time during swallowing between the with and without cue conditions, because the most frequent coordination type exhibited no difference between the with and without cue conditions. Although most previous studies have reported that the duration of expiratory phase is lengthened during swallowing, they all included swallowing apnoea. In this study, however, we excluded this period in the calculations. Paydarfar et al. (1995) investigated the relationship between respiration and swallowing dynamics in humans. They studied three types of swallowing tasks: spontaneous swallowing, evoked swallowing by water injection, and cued swallowing. The authors found that the smaller the time interval between the onset of the expiratory phase and the onset of the Supra EMG burst, the longer the time between the onset of the Supra EMG burst to the onset of the inspiratory phase after swallowing, without any effects on the duration of swallowing apnoea, in all swallowing tasks. Since swallowing was recorded only once in each condition in this study, we were unable to clarify how the timing of swallowing initiation affected the subsequent phase. However, it should be noted that there was a significant difference in the whole duration of the expiratory phase between the with and without cue conditions. Changes in duration in the expiratory phase without swallowing apnoea were smallest in the without cue condition. Thus, we speculate that voluntary swallowing with cue may modulate the coordination of respiration and swallowing by lengthening the expiratory phase duration.

### Coordination of Respiration and Swallowing During 100-ml Water Swallowing

All but five participants successfully completed the 100-ml water swallowing task with breath-holding. As expected, the most frequent coordination pattern was EE type, as in the 3-ml water swallowing task. Therefore, we evaluated how sequential 100-ml swallowing affected the coordination of respiration and swallowing in EE type.

In animal studies, high-frequency repetitive swallowing has been reported to be evoked by continuous electrical stimulation applied to the superior laryngeal nerve (Fukuhara et al., 2011; Tsuji et al., 2014; Suzuki et al., 2018; Yoshihara et al., 2020) or capsaicin application to the vocal folds (Tsujimura et al., 2016, 2017; Tsuji et al., 2020). In these cases, both the rhythm and amplitude of breaths are reported to be increased after cessation of repetitive swallows (Doty, 1968; Dick et al., 1993). However, the findings of these animal studies are not comparable to the results of this study because the animals were anesthetized and did not control the rhythmicity or swallowing movements by themselves. Numerous previous studies have investigated how sequential swallowing affects respiration pre- and post-swallowing in humans (Dozier et al., 2006; Daniels et al., 2007; Wheeler Hegland et al., 2011; Lederle et al., 2012). However, the respiratory pattern at pre- and post-swallowing has varied between previous studies. For example, the percentage of occurrence of the expiratory phase immediately before and after sequential swallowing (i.e., the EE type) was reported to be 33% for 100 ml (Wheeler Hegland et al., 2011) and 38.6% for 50 ml (Dozier et al., 2006), while it was 52.4% (11/21 participants) in this study.

Since the duration of swallowing apnoea was long and the respiration was inhibited during repetitive swallowing, the respiratory drive could be facilitated, at least after swallowing. Based on this perspective, we recorded several respiratory cycles before and after swallowing. The duration of the expiratory phase immediately before swallowing was smaller than that following swallowing, whereas the duration of the inspiratory phase remained unchanged. Although we did not measure oxygen saturation and tidal volume, it could be assumed that breath-holding for several seconds during 100-ml water swallowing facilitated respiratory drive. Strohl and Altose (1984) observed a decrease in oxygen saturation during 10–20-s breath-holding, although the rate of fall of oxygen saturation was relatively small. Decreased oxygen saturation leads to a decrease in oxygen partial pressure as well as an increase in carbon dioxide partial pressure. Changes in respiratory frequency have been reported under experimental conditions, such as hypoxia or hypercapnia (Rebuck et al., 1976; Gardner, 1980; Cunningham and Robbins, 1984; Dozier et al., 2006). Rebuck et al. (1976) reported that the inspiratory time remained constant during hypercapnia until tidal volume had increased during hypercapnia, consistent with the results of this study. However, the authors found that the respiratory rate increased with shortening expiratory time when the tidal volume increased 3–5 times of the eupneic value. Jennett et al. (1981) reported that following a brief hypoxic stimulus, frequency changes resulted from alterations in the two phases; when total respiratory time decreased, it was always linked to a decrease in expiratory time, and an increase in the respiratory cycle time was due to an increase in the inspiratory time. In this study, it can be assumed that changes in respiratory rhythm were not due to hypoxia or hypercapnia but can be attributed to voluntary behaviors to maintain the oxygen level in the lungs. Most of the participants understood how to swallow a large amount of water (100 ml) with breath-holding. Thus, the participants would be expected to aim to maintain a sufficient oxygen level to hold their breath during swallowing. We did not find evidence of strong inspiratory effort but observed a significantly shortened duration of the expiratory phase before swallowing compared with that after swallowing. Future studies should measure tidal volume, including expired and inspired volume, to clarify how respiratory effort is affected by sequential swallowing.

### Coordination of Respiration and Swallowing During Chewing

The results revealed that the most frequent coordination pattern was EE type during chewing, in accord with previous studies (Mead and Reid, 1988; McFarland and Lund, 1995; Matsuo et al., 2008). Mechanisms of swallowing initiation at the expiratory phase during chewing different from those in a single or sequential swallow should be considered. During chewing, a person does not determine the timing of swallowing by themselves. In this process, the triturated food bolus is transported into the mid-pharynx, where the bolus is accumulated (Palmer et al., 1992). Once the pharyngeal mucosa, which is innervated by the pharyngeal branch of the vagal nerve or the superior laryngeal nerve, is stimulated by the bolus, the swallowing reflex is readily evoked. Swallowing initiation would be expected to be determined dominantly by the bolus condition, such as the location or texture, rather than the respiratory phase. Matsuo et al. (2008) reported that pharyngeal bolus aggregation started 1.27 s before the end of the inspiratory phase and lasted until the end of this phase, which was followed by the expiratory phase and swallowing. Mead and Reid (1988) found expiratory muscle activation when airflow was interrupted at the glottis. It is likely that bolus propulsion initiates expiration followed by the swallowing reflex. Further, during pharyngeal swallowing, when hyolaryngeal elevation contributes to passive opening of upper oesophageal sphincter, infrahyoid muscles, such as the thyrohyoid, omohyoid, sternohyoid, and sternothyroid muscles, are also activated. These are all inspiratory muscles and are activated synchronously with the diaphragm (Mitchinson and Yoffey, 1947; Megirian et al., 1985). During the inspiratory phase, contraction of the diaphragm caused pulling of the trachea and larynx inferiorly, and the contraction of all infrahyoid muscles resulted in pulling down of the hyoid and thyroid cartilages, which have antagonistic functions for swallowing. Thus, it is possible that bolus stimulation applied to the pharyngeal and laryngeal region activates expiratory muscle activity and then initiates the swallowing reflex during the expiratory phase.

The effects of mastication on respiratory function have not yet been clarified; although one study reported no effect (Smith et al., 1989). Other studies reported a decrease in respiratory cycle time (Fontana et al., 1992; McFarland and Lund, 1995; Matsuo et al., 2008). The results are consistent with the latter finding and reveal that the respiratory cycle time, as with the expiratory and inspiratory time, significantly decreased during chewing. In addition, there was a significant difference in the expiratory time between the first and last respiration before swallowing.

We initially predicted the spatial interruption of the upper airway by the bolus during chewing. During chewing, dynamic movements of the tongue body are needed not only in the anterior but also in the posterior region, which was observed by a VF study (Palmer et al., 1997; Hiiemae et al., 2002). Since the posterior tongue forms the anterior wall of the mid-pharynx, tongue movements might be expected to interfere with respiration by narrowing the airway in the pharynx. This was, however, not the case in this study, as the results revealed no significant differences in the width or A–P distance of the pharynx between the control and chewing conditions.

It is well documented that exercise causes increased respiratory function. Exercise may cause metabolic demand, which causes hyperpnea. Although mastication is not a kind of exercise but can be considered a natural behavior in human life, chewing has been found to decrease the respiratory cycle time depending on the chewing speed (Formiga et al., 2005), and to decrease the tidal volume with an increase in respiratory frequency (Fontana et al., 1992; McFarland and Lund, 1995). Furthermore, as previously reported, changes in respiratory cycle time can result from alterations either when total respiratory time decreased with a decrease in expiratory time or increased respiratory cycle time with an increase in the inspiratory time under hypoxic conditions (Jennett et al., 1981). We speculate that the decrease in respiratory cycle time and gradual decrease throughout the chewing process may have been due to changes in metabolic demand.

It is possible that the pattern of respiration was irregular during chewing, which could also have important implications. In this study, there was no relationship between the respiratory cycle time between at rest and during chewing in each individual. The pattern of breathing is regulated by the respiratory CPG, so that normal ventilation can be achieved with the lowest work output or energy expenditure. Performing a chewing task may cause deviation from this optimal pattern, and, hence, an increase in the work of breathing. Thus, irregularity in the modulation of respiratory function may be associated with individual dependent increased loads.

Five participants exhibited markedly inhibited respiration during chewing, in accord with the findings of previous studies (McFarland and Lund, 1995; Matsuo et al., 2008). Although we were not able to clarify the mechanisms underlying this finding, it is possible that the result was caused by the experimental conditions. In this study, a videoendoscopic fiber was inserted into the nasal cavity of the participants on either side, which may have interfered with nasal air flow during chewing. Future studies should clarify whether the inhibition was caused by this condition in some participants, or if it represents an exaggerated response, as suggested by Matsuo et al. (2008).

### Clinical Implications

Understanding the coordination of respiration and swallowing is particularly important in clinical situations. For example, respiratory impairment is defined as an age-related reduction in ventilatory control, and weakened respiratory muscle strength and respiratory mechanics (Chen and Kuo, 1989; Enright et al., 1994; Sachs et al., 2009), as well as ineffective gas exchange (Vaz Fragoso and Gill, 2012). Parreira et al. (2010) suggested that thoracoabdominal motion, rather than breathing pattern, was affected by age. Furthermore, older people have been reported to exhibit reduced responses to hypoxia or hypercapnia compared with young people (Kronenberg and Drage, 1973; Peterson et al., 1981). Previous studies have reported an effect of aging on swallowing and the coordination between respiration and swallowing (Selley et al., 1989; Robbins et al., 1992; Sonies, 1992; Wang et al., 2015). Wang et al. (2015) reported that older people exhibited delayed onset latency of the swallowing reflex and longer swallowing apnoea duration, which depended on the bolus volume. The authors also noted that older people exhibited less EE-type swallowing during dry and water swallowing compared with young people. In this study, the results revealed that command swallowing in response to a cue affected expiratory duration. If pulmonary function is impaired in patients, collapse of coordination may be associated with a risk of bolus penetration or aspiration. In addition, impaired chewing function may lead not only to longer chewing duration but also to collapse of coordination among chewing, respiration, and swallowing. Clinicians should pay attention to swallowing in patients with stroke, Parkinson’s disease, and any other conditions that potentially cause swallowing disorders (Wirth et al., 2016). Future studies should investigate how aging itself or diseases affect swallowing function and coordination between respiration and swallowing.

### Limitations

Several limitations should be considered when interpreting the findings of this study. First, we recruited only 21 healthy participants. In a future study, it would be useful to evaluate how aging or other personal factors affect not only swallowing but also coordination between respiratory and swallowing functions. Second, we recorded only 3- and 100-ml water swallowing as well as solid food chewing and swallowing in a pre-determined order. Bolus volume or order may affect the coordination between respiration and swallowing. Third, we measured nasal temperature to monitor respiration because this method was convenient and easy for combining the data for analysis. Plethysmography is a system for measuring respiratory function, enabling three-dimensional assessment of absolute chest wall volumes. Matsuo et al. (2008) concluded that plethysmography was better than nasal manometry for determining the respiratory phase during chewing and swallowing. Future studies should clarify tidal volume and pulmonary ventilation using plethysmography. Finally, we only calculated pharyngeal volume by two-dimensional measurement. The methods of this study should be validated in future studies using VF or three-dimensional computed tomography images.

### Conclusion

The major coordination pattern of respiration and swallowing was EE type during 3-ml water swallowing, either with or without cue, and during 100-ml water swallowing and chewing. Although cueing did not affect swallowing movements, the expiratory time was lengthened by the cue. During 100-ml swallowing, the respiratory cycle time and expiratory time immediately before swallowing were significantly smaller than those during and after swallowing, while the inspiratory time did not differ throughout the recording period. During chewing, the respiratory cycle time was decreased in a time-dependent manner, probably because of metabolic demands. The coordination of the two functions is maintained not only in voluntary swallowing but also in involuntary swallowing during chewing. Understanding the mechanisms underlying respiration and swallowing is important for evaluating the coordination required to complete safe swallowing in older people and patients with dysphagia.

## Data Availability Statement

The original contributions presented in the study are included in the article/Supplementary Material, further inquiries can be directed to the corresponding author.

## Ethics Statement

This study was approved by the Ethics Committee of the Niigata University (2020-0131). The patients/participants provided their written informed consent to participate in this study.

## Author Contributions

NH and MI were involved in the conception and design of this study. NH, AS, SK, YN, KN, JM, TT, and MI were involved in the acquisition of the data, and drafting and critical revision of the study for important intellectual content. NH, AS, and MI were involved in the analysis or interpretation of the data. All authors approved the final version of the manuscript submitted for publication and agreed to be accountable for all aspects of the work in ensuring that questions related to the accuracy or integrity of any part of the study are appropriately investigated and resolved.

## Conflict of Interest

The authors declare that the research was conducted in the absence of any commercial or financial relationships that could be construed as a potential conflict of interest.
