# Supplementary material for: Coordination of Respiration, Swallowing, and Chewing in Healthy Young Adults
Source: Front Physiol. 2021 Jul 13;12:696071. doi: 10.3389/fphys.2021.696071 (PMC8313873; doi:10.3389/fphys.2021.696071)
Supplement: Supplementary file 1 [file Data_Sheet_1.DOCX]

Supplementary Material

**Supplementary Figure 1.** Changes of nasal temperature. Increasing rate of nasal temperature during breath-holding was significantly negatively correlated with the initial temperature, although the change was small. Regression formula, Pearson’s product-moment correlation coefficient and P-value were y = −0.0598x + 1.7398, R² = 0.9947 and P < 0.001, respectively.

**Supplementary Figure 2.** Videoendoscopic images of the pharynx at rest and during chewing. The width of lateral distance (Width) and A-P distance of the pharynx were measured on the view and normalized to the width of epiglottis. A, transition from inspiration to expiration at rest; B, transition from expiration to inspiration at rest; C, third peak of masseter EMG burst during chewing; D, third peak time of suprahyoid EMG burst during chewing.

**Supplementary Figure 3.** Effect of chewing on the size of pharynx. There was no difference in the width (left) and A-P distance of pharynx (right) among the conditions (one-way repeated measures ANOVA). Rest (IE), transition from inspiration to expiration at rest; Rest (EI), transition from expiration to inspiration; 1st Mas and Supra, third peak of masseter and suprahyoid EMG bursts during chewing, respectively; 2nd Mas and Supra, last third peak of masseter and suprahyoid EMG bursts during chewing, respectively.
